# Social cybersecurity: an emerging science

**DOI:** 10.1007/s10588-020-09322-9

**Published:** 2020-11-16

**Authors:** Kathleen M. Carley

**Affiliations:** Center for Informed Democracy and Social Cybersecurity, Carnegie Mellon University, Pittsburgh, PA USA

**Keywords:** Social cybersecurity, Social network analysis, Dynamic network analysis, Social media analytics, Review

## Abstract

With the rise of online platforms where individuals could gather and spread information came the rise of online cybercrimes aimed at taking advantage of not just single individuals but collectives. In response, researchers and practitioners began trying to understand this digital playground and the way in which individuals who were socially and digitally embedded could be manipulated. What is emerging is a new scientific and engineering discipline—social cybersecurity. This paper defines this emerging area, provides case examples of the research issues and types of tools needed, and lays out a program of research in this area.

In today’s high tech world, beliefs opinions and attitudes are shaped as people engage with others in social media, and through the internet. Stories from creditable news sources and finding from science are challenged by actors who are actively engaged in influence operations on the internet. Lone wolfs, and large propaganda machines both disrupt civil discourse, sew discord and spread disinformation. Bots, cyborgs, trolls, sock-puppets, deep fakes, and memes are just a few of the technologies used in social engineering aimed at undermining civil society and supporting adversarial or business agendas. How can social discourse without undue influence persist in such an environment? What are the types of tools and theories needed to support such open discourse?

Today scientists from a large number of disciplines are working collaboratively to develop these new tools and theories. There work has led to the emergence of a new area of science—social cybersecurity. Herein, this emerging scientific area is described. Illustrative case studies are used to showcase the types of tools and theories needed. New theories and methods are also described.

## Social cybersecurity

In response to these cyber-mediated threats to democracy, a new scientific discipline has emerged—social cybersecurity. As noted by the National Academies of Science NAS (2019): Social cybersecurity is an applied computational social science with two objectives

- “characterize, understand, and forecast cyber-mediated changes in human behavior and in social, cultural, and political outcomes; and
- build a social cyber infrastructure that will allow the essential character of a society to persist in a cyber-mediated information environment that is characterized by changing conditions, actual or imminent social cyberthreats, and cyber-mediated threats.”

Social cybersecurity is both a new scientific and a new engineering field. It is a computational social science with a large foot in the area of applied research. Drawing on a huge range of disciplines the new technologies and findings in social cybersecurity have near immediate application on the internet. The findings and methods are relevant to policy makers, scholars, and corporations.

Social cybersecurity uses computational social science techniques to identify, counter, and measure (or assess) the impact of communication objectives. The methods and findings in this area are critical, and advance industry-accepted practices for communication, journalism and marketing research. The field itself has a theory, application, and policy component. The methods build on work in high dimensional network analysis, data science, machine learning, natural language processing and agent-based simulation. These methods are used to provide evidence about **who** is manipulating social media and the internet for/against you or your organization, **what** methods are being used, and **how** these social manipulation methods can be countered. They also support cyber diplomacy (Goolsby 2020).

Social cybersecurity uses computational social science techniques to identify, counter, and measure (or assess), the impact of influence campaigns, and to identify and inoculate those at risk against such campaigns. The methods and findings in this area are critical, and advance practices for intelligence and forensics research. These methods also provide scalable techniques for assessing and predicting the impact of influence operations carried out through social media, and for securing social activity on the internet and mitigating the effects of malicious and undue influence. As such they are critical for creating a more secure and resilient society.

Influence campaigns vary widely, and who is at risk in part depends on those conducting the influence campaign and in part on the context. For example, in our research we found that influence campaigns appearing to come from state-level actors during the elections in Western Europe and the US from 2016 to 2020 were often aimed at minorities. For example, they targeted women, ethnic minorities, and the LGBQT community. In contrast, in India as COVID-19 ramped up internal non-state groups launched anti-Muslim campaigns. As movies like the Black Panther and Captain Marvel were released individual’s launched campaigns against the movies. In the elections in the Asia Pacific region influence campaigns often take the form of promoting pro-China candidates. Many influence campaigns are aimed at specific individuals trying to recruit them to a new cause, or engage them in insider threat activity.

Social cybersecurity is distinct from cybersecurity. Cybersecurity is focused on machines, and how computers and databases can be compromised. In contrast, social cybersecurity is focused on humans and how these humans can be compromised, converted, and relegated to the unimportant. Where cybersecurity experts are expected to understand the technology, computer science, and engineering; social cybersecurity experts are expected to understand social communication and community building, statistics, social networks, and machine learning. Social cybersecurity is also distinct from cognitive security. Cognitive security is focused on human cognition and how messages can be crafted to take advantage of normal cognitive limitations. In contrast, social cybersecurity is focused on humans situated in society and how the digital environment can be manipulated to alter both the community and the narrative. Where cognitive security experts are expected to understand psychology, social cybersecurity experts are expected to have a broader social science expertise.

**Fig. 1:**
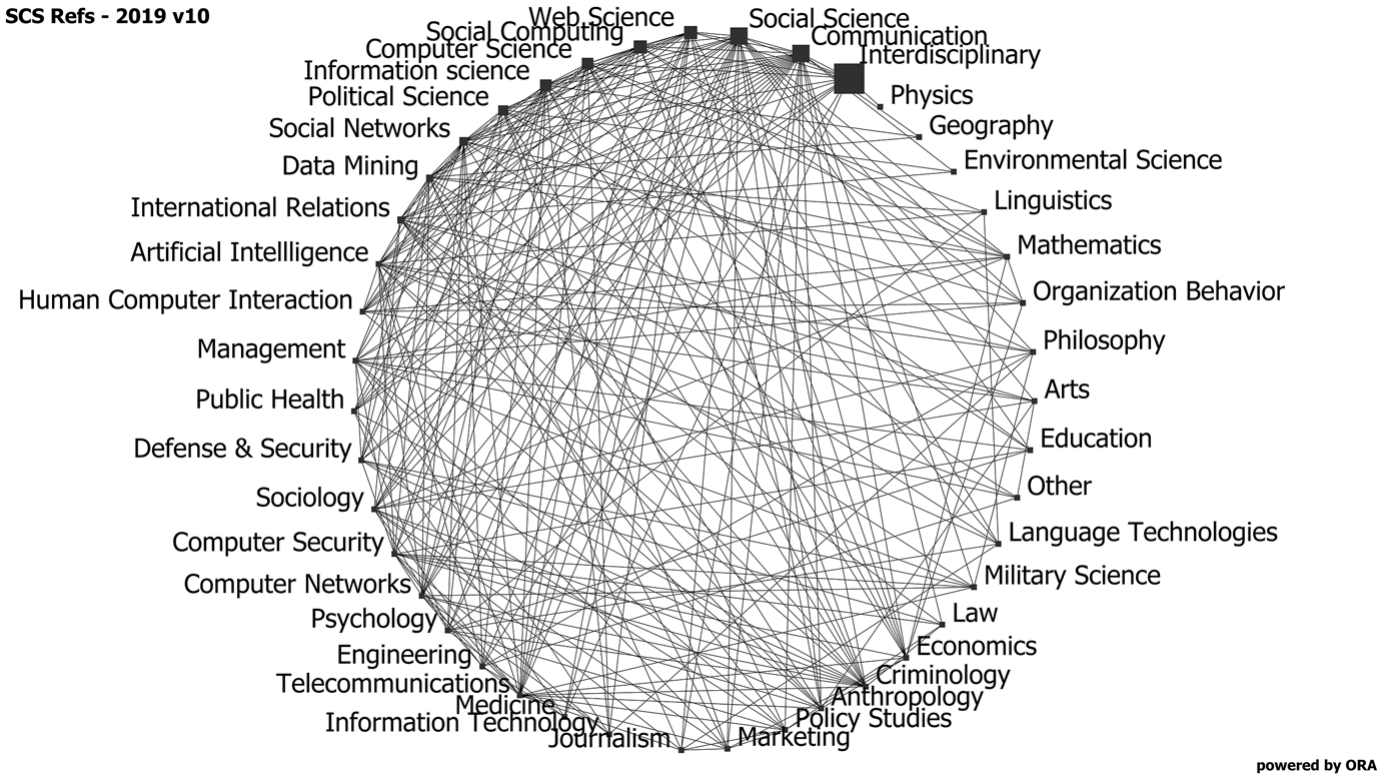
Network diagram of the interdisciplinary nature of the field of social cybersecurity. Nodes are disciplines and are sized by number of articles. Links are number of articles associated with both disciplines

In our research we have found that there is some work in social cybersecurity that draws on most scientific fields. In a recent study of the field, we identified 1437 papers up through 2019. Each journal was coded by the dominant scientific fields that it is associated with. The results was a set of 43 disciplines. In Fig. 1 we show the discipline to discipline network where the links indicate the number of articles that draw on both disciplines. The size of the nodes indicate the number of articles associated with that discipline.

As seen in Fig. 1, social cybersecurity is very much a computational social science, with a strong interdisciplinary focus drawing on the social and communication and computer sciences. The dominant methods are social network analysis, data mining, and artificial intelligence (which includes language technologies and machine learning). The areas that have dominated research in this area are largely ones that draw on theories from diverse disciplines.

Within social cybersecurity artificial intelligence is coupled with social network analysis to provide new tools and metrics to support the decision maker. Recent research in social cybersecurity is enabling new tools to support research methodology and metrics-based decision making for communicators. The following case studies highlight the types of research findings made possible in the area of social cybersecurity using these new tools. After presenting these case studies we then turn to a discussion of a new orienting approach for doing research in social cybersecurity, referred to as the BEND framework. Collectively, these items provide a glimpse into the core of this emerging scientific field.

**Fig. 2:**
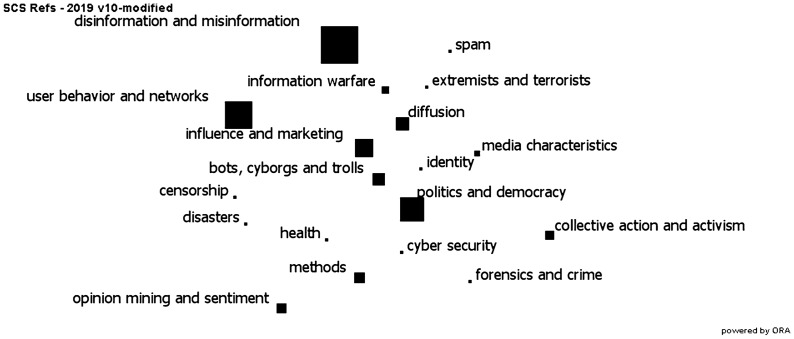
Research topic areas in social cybersecurity

What are the dominant themes in social cybersecurity? As can be seen in Fig. 2, the dominant research area currently is disinformation. This is followed by research on user behavior and networks on the web, and then research on politics and democracy. In Fig. 2 each node represents a research topic and the size of the node reflects the number of articles on that topic. There are two caveats, first, the size of the disinformation node is growing rapidly with all the new papers related to COVID-19 and the elections. Second, the reader may wonder why privacy does not appear. This is because privacy was viewed as a separate field unto itself and papers in that area were not include in the analysis.

## Case study 1: building community in social media

In Ukraine there were a group of young men sending out provocative images of women. They didn’t know each other they were just posting images they liked. Bots were used in an influence campaign to send out tweets mentioning each other and multiple of these young men at once. This led the men to learn of others who, like them, were sending out these images. They formed an online group—a topic oriented community. Once formed, the bots now-and-then tweeted information about where to get guns, ammunition, and how to get involved in the fight. Why did this work?

The cyber landscape is populated by topic oriented communities—groups of actors all communicating with each other about a topic of interest. Each actor can be in many topic oriented communities. Actors can be people, bots, cyborgs (person with bot assistance), trolls (person seeking to disrupt, a corporation or government account using a fake persona and often engaging in hate speech and identity bashing), and so forth. Members of a topic oriented community are loosely connected by the fact that they interact with each other. For example, they might friend, follow, retweet, mention, reply, quote or like each other. Some actors will be opinion leaders, some will have a disproportionate ability to get messages to the community (super-spreaders), some will be highly involved in the mutual give and take of an on-going discussion (super-spreaders), some will just be lurking on the sidelines. The members of topic oriented communities are also loosely connected because they are sending or sending or receiving messages about the same topics. For example, they are all discussing the Army-Navy game. Some actors will be more actively engaged and send more messages. Topic oriented communities range in size and how they are organized; e.g. plane spotters is a vast community that is only slightly connected. Through new tools and research methodologies that measure communication impacts via social media, it is now possible to measure and visualize data to demonstrate topic oriented communities that become overly connected become echo-chambers. (Note: An echo-chamber is a pathologic form of a topic oriented community in which the level of connection is extremely dense and the topic highly shared and narrow.) Messages sent within echo-chambers reach all quickly, and such groups can often be readily excited to respond emotionally rather than rationally to outside information.

For Ukraine bots were used to send communications which basically introduced these young men to each other through the use of @mentions. These bots also sent provocative images. The young men then began to follow each other forming a topic oriented community. In Ukraine influencers created/controlled the bots that conducted a “build” campaign to misinform (engendering social connections between the young men by mentioning them together). At the same time, they conducted an “enhance” campaign by rebroadcasting some images and pointing to others and an “excite” campaign with new positive language. Once the group was established, a “distort” campaign appeared bringing in information relative to the revolution.

For additional details on this case study see (Benigni et al. 2019).

## Case study 2: increasing communicative reach in social media

Syrian expats and sympathizers with ISIS were engaged in social media conversations. This included listening to the preaching’s of a prominent Imam. A group of actors infiltrated this group and redirected attention to a site collecting money for the children of Syria. How was this done?

In social media, your followers may not receive your messages, or your messages may not be prioritized so that they appear prominently to those concerned with your messages. Social media platforms use your social network position (how you are connected to others), and the content of your message, to decide who to recommend your message to, when, and in what order. Who you mention in posts, which hashtags you use, whether you use memes or link to YouTube videos, the frequency with which you post, the number of others who follow you or like your posts, all impact whether your message is prioritized.

In this Syrian ex-pat community influencers created/controlled a social influence bot, the Firibi gnome bot. This set of bots was used to conduct a sophisticated influence campaign. Multiple copies of this bot were released which proceeded to send out messages mentioning each other and so engaged in a “build” campaign to misinform. The results was a topic oriented community of bots, which meant that messages from any one bot would be recommended to others interested in similar topics. Then these bots started following retweeting messages from an Imam, who may not have been aware of this activity. This “boosted” the social influence of the Imam, and “engaged” the bot with the community. Since the Imam was a super-spreader, this also meant that messages from the Firibi gnome would be prioritized to the Imams followers. Then, the Firibi gnome bot engaged in an “enhance” campaign and started sending messages recommending the charity website. This message was then prioritized.

For additional details on this case study see (Benigni et al. 2017).

## Case study 3: conspiracies in social media

As COVID-19 spread so to dis disinformation regarding the pandemic. Thousands of disinformation stories were spread focusing on false cures and prevention techniques, false characterization of government response, and claims of leaders having COVID even when they did not. Throughout a number of conspiracy stories appeared and began to gain traction. How was this done?

In social media, messages gain traction through amplification and saturation. A message from a single actor or site can be amplified and spread by bots and trolls. This means those messages will get shared disproportionately and may even be made to trend. The more shared, the more the story gets prioritized to individuals who do not pay attention to the original source but may be following users who follow those who follow the source. Further, in social media the presence of a story on two or more platforms does not mean that the story has been independently validated. Indeed stories often appear on one platform, and bots and trolls are used to push it to other platforms. Marketing firms hired to spread disinformation can place the same story simultaneously in different forms on each of the platforms. Twitter, e.g., is often used to garner attention to YouTube videos and to promulgate stories that first appear in Blogs or on Facebook. The more platforms a story appears on, the more it saturates the digital space, the more real it seems, particularly when it is accompanied by images and videos and supported by celebrities and authorities.

A number of conspiracies surrounded COVID-19. One is that the virus was created in a US lab and carried to Wuhan by US soldiers engaged in a war game, and another is that it was created by Bill Gates and then spread as step 1 in a plan to create a new world order. Stories regarding some of these conspiracies appeared on Chinese state sponsored media. Bots surrounding these media then retweeted the stories—thus amplifying the reach. Related stories and even a “plandemic” video were released on multiple media e.g., Facebook, YouTube, Twitter and Instagram. Bots further amplified these messages or sent messages with the related URLS. Trolls, employed hate speech to denigrate those that tried to counter the conspiracy messages, were implicated by the conspiracy messages, or that were anticipated to not believe the conspiracy messages. The same conspiracy stories, often providing additional details, also appeared on the purported “fake news” news-sites which are websites that purport to be news agencies but either are not news sites or have dubious editorial procedures and are known for spreading disinformation. Large numbers of bots surround these sites and would send out messages with URLS to these sites. Even larger numbers of bots retweeted messages referencing these sites. The result was a topic oriented community of conspiracy theorists, bots, and trolls around various conspiracy thrusts, an increase in the number of conspiracies that were spreading, the re-appearance of conspiracy stories even if they were banned, and increasingly elaborate conspiracies such as the new world order.

For additional details on this case study see (Huang, 2019).

## The BEND framework

The foregoing case studies indicate the types of issues that need to be considered by a social cybersecurity researcher or practitioner. They also suggest the need for new technologies such as those to identify disinformation, bots, trolls, cyborgs and memes. Finally they point to the need for new theories to make sense of the way in which influence plays out in social media. One such transdisciplinary theory is referred to as the BEND framework.

Influence campaigns are often described in terms of the 4Ds—distract, distort, dismay and disrupt (Nimmo, 2015). These are often used to describe information operations by Russia. However, as the three case studies illustrate influence operations don’t involve just messages with distract, distort, dismay or disrupt campaigns. Our research suggests that a broader understanding of information maneuvers is needed. Specifically, information campaigns that are successful typically impact both community and narrative. That is maneuvers are conducted that alter both who is communicating with whom as well as what is being communicated. Further, the 4Ds are essentially negative maneuvers; that is, they are problem creation not problem solution maneuvers. In the Ukraine—images were used to excite, in Syria—messages were used to explain, in COVID-19 explain and enhance messages were used. While in each case these are not the only kind of messages used, the point is, there was more than just the 4Ds. The.

BEND framework argues that influence campaigns are comprised of sets of narrative and structural maneuvers, carried out by one or more actors by engaging others in the cyber environment with the intent of altering topic-oriented communities and the position of actors within these communities. A topic oriented community is a group of actors who are more or less talking to each other about more or less the same thing. This engagement with the topic oriented community is often assisted by or carried out by bots, trolls and cyborgs in addition to human users. This engagement is aimed at manipulating either or both the narrative (what is being talked about) and the community (who is talking to whom). Bots, trolls, cyborgs and humans engage with others in cyberspace in ways designed to, and send messages that are constructed to, take advantage of three things: the technology, the mind and emotions, and the world view. Technology: these activities are designed to exploit the algorithms that prioritize the order in which messages are presented and the recommendation algorithms so that messages selected by the perpetrator appear first, often, and trend, and the goods, services, urls, and actors they mention are recommended to the readers. Mind & Emotion: these activities are designed to exploit natural human biases and reflexes such as confirmation bias, escalation of commitment, and the fear or flight reflex. World View: these activities are designed to make use human social cognition, the set of heuristics we use to make sense of vast quantities of data in terms of group—such as the generalized other and stereotyping.

In social cybersecurity, theories and methods go hand-in-hand. Hence associated with the BEND theory is a methodology for empirically assessing which maneuvers are being used and for measuring the impact of social media communication research, planning, and objectives (Beskow and Carley 2019). The BEND framework thus has associated with it a set of methods and tools for looking at who engaged in what information maneuvers directed at whom with what impact. The BEND framework characterizes communication objectives and so the maneuvers into 16 objectives such that 8 are aimed at shaping the social networks of who is communicating with whom and 8 are aimed at shaping the narrative. For the social network, there are four positive objectives (the four B’s) and four negative objectives (the four N’s). Similarly, for shaping the narrative there are four positive objectives (the four E’s) and the four traditional negative objectives (the four D’s). These are described in Table 1.

**Table 1:** Communication objectives BEND

	Manipulating the narrative		Manipulating the social network	
Positive	**Engage**	Messages that bring up a related but relevant topic	**Back**	Actions that increase the importance of the opinion leader or create a new opinion leader
	**Explain**	Messages that provides details on or elaborate the topic	**Build**	Actions that create a group or the appearance of a group
	**Excite**	messages that elicit a positive emotion such as joy or excitement	**Bridge**	Actions that build a connection between two or more groups
	**Enhance**	Messages that encourage the topic-group to continue with the topic	**Boost**	Actions that grow the size of the group or make it appear that it has grown
Negative	**Dismiss**	Messages about why the topic is not important	**Neutralize**	Actions decrease the importance of the opinion leader
	**Distort**	Messages that alter the main message of the topic	**Nuke**	Actions that lead to a group being dismantled or breaking up, or appearing to be broken up
	**Dismay**	Messages that elicit a negative emotion such as sadness or anger	**Narrow**	Actions that lead to a group becoming sequestered from other groups or marginalized
	**Distract**	Discussion about a totally different topic and irrelevant	**Neglect**	Actions that reduce the size of the group or make it appear that the group has grown smaller

The BEND framework is the product of years of research on disinformation and other forms of communication based influence campaigns, and communication objectives of Russia and other adversarial communities, including terror groups such as ISIS that began in late December of 2013. It draws on both findings regarding the communication objectives and tactics of adversarial actors adversarial (Benigni et al. 2017; Lucas and Nimmo 2015; Manheim 1994), political influence (Howard and Kollanyi 2016; Howard et al. 2018; Huckfeldt and Sprague 1995), marketing (Webster 2020), psychology (Sanborn and Harris 2013). The BEND Framework addresses these communication objectives and tactics from a transdisciplinary perspective. Early evidence suggests that excite, enhance, dismay, and distort may be the most common communication objectives used to spread disinformation.

The BEND framework is more than a description of the maneuvers shown in Table 1. It begins with identifying the types of user who is conducting a maneuver or is targeted by such a maneuver. Actors are characterized by whether they are bots, trolls, news agencies, government actors, celebrities or are influential super-friends (those with a high number of reciprocated ties in social media), super-spreaders (those with a high number of others who they reach with their messages e.g. a large number of followers etc.) or are otherwise influential in social media (e.g. send a large number of messages). Actors being targeted can be topic oriented communities or individual actors. Then each of the messages are characterized for which of the 16 maneuverers the message is consistent with using a set of discreet measures. Finally, impact in terms of change in the target is assessed from a social network perspective and content perspective exploring how over time the target has changed.

Associated with the BEND maeuvers are a series of measures and indicators for each of the objectives. These have been operationalized, are now part of the ORA-PRO social media tools, and have been tested on Twitter data. They were used in assessing data during various NATO exercises, Naval exercises, elections, and disasters. We find that in many cases complex influence campaigns involve using multiple BEND objectives as was described in the three case studies.

## Using social network analysis and artificial intelligence

One of the key tools in social cybersecurity is high dimensional dynamic social network analysis. Social network analysis is the analysis of who interacts with whom. Network techniques have long been used in intelligence for identifying groups and tracking adversarial actors and by marketers for identifying key informants and opinion leaders. With social media such techniques have been expanded to enable scalable solutions for massive data that take into account multiple types of relations among actors as well as relations among resources, ideas and so forth. Today such high dimensional dynamic network techniques underlie social media analysis. The two interaction networks—such as (1) who likes or retweets whom and (2) the content of the messages—are treated as networks. The techniques to identify these interactions are embedded in ORA-PRO and are used for identifying topic-groups and the influential actors within these groups; the depth of this data is not possible with other off-the-shelf analysis tools. Running social network techniques on social media provides indicators that can then be used in machine learning tools to identify actors and messages of interest such as bots, cyborgs, and trolls.

Artificial intelligence (AI) techniques, particularly machine learning and natural language processing techniques are also key tools in social cybersecurity. AI, and particularly machine learning (ML), are often pointed to as force multipliers in dealing with the vast quantity of digital data available today. Such technologies are clearly of value; however, they are not the panacea envisioned. The problems faced by the military in social cyberwar are continually changing and often occur only once; thus, new technique for responding are continuously needed. Further, current AI and ML techniques are often focused on easily measured data rather than the more volatile socio-political-cultural context.

Language technologies are used for translation, sentiment, and stance detection. Most sentiment tools simply inform the reader if a message containing a word of interest is positive or negative, which often has no relation to the sentiment about the word of interest. We find that as much as 50% of the time the sentiment toward the word of interest is the opposite as the sentiment of the message as a whole. Consider the sentiment toward U.S. in the message—“I hate Russian interference in social media and treatment of the U.S. as evil”. The sentiment of the message as a whole is negative; but it is positive toward the U.S.. In contrast, the NetMapper system used with the BEND framework identifies the sentiment about the word of interest, and measures a set of subconscious CUES in the message to assess the sender’s emotional state.

Machine learning techniques are frequently used to identify bots, false statements, and message on particular topics. An example is BotHunter. Such tools can rapidly identify the likelihood that potential actors are Bots. This can indeed support analysis and help a communicator understand an adversary’s communication objectives. However these, tools that are based on “supervised” learning have a limited shelf life. First, they require large training sets. Training sets need to be created by humans tediously coding messages and the senders of messages into categories required for the AI tool. Today, bots are evolving faster than are the tools to find them in large part because it takes too long to create training sets. Training sets are often biased—e.g., sentiment training sets are biased toward lower middle-class ways of expressing sentiment in English. The AI tools themselves give probability scores and no explanation on why they reached the conclusion they did. Bot detection tools often disagree because the tools were “trained” differently—leaving the ultimate decision in the hands of the analyst. These factors reduce how long these technologies will be useful for and in what contexts. Today’s technology advances are being made in developing AI techniques that do not require massive training sets and that provide explanations—BotRecommender is such a tool.

For disinformation, the issues are legion and there are many types of disinformation as is illustrated in Table 2. Fact-checking tools using humans or human-AI teams are providing valuable guidance but so far take a long time to determine if a story contains an inaccuracy. Assessing intent is difficult—was the sender intentionally trying to deceive (disinformation) or were they just mistaken (misinformation). Many disinformation campaigns are not based on inaccurate facts, but on innuendo, fights of illogic, reasoning from data taken out of context, and so on. Many times, stories labeled as disinformation are simple alternative interpretations of facts. AI only helps for some types of disinformation. It is less useful the more unique the storyline, and the faster the story spreads.

**Table 2:** Types of “disinformation”

Disinformation types	Example	Potential for AI techniques to detect
Fake news (story made to look like news)	Naval Destroyer crash in Hurricane Harvey	AI could be used to identify sites, and do fact checking
Fabrication with visual	Parkland student ripping up constitution	AI could be used to create and identify fake images
Fabrication without visual	Opposition peso scam in Philippines	AI might be of some assistance in finding all instances of story
Propaganda	Duerte’s helicopter scaring off the Chinese	AI could help classify underlying BEND objectives
Conspiracy	Pizzagate	AI could be used to do fact checking
Misleading—due to misquoting	Captain Marvel—Brie Larson is a racist/sexist	AI could be used to do fact checking, and stance checking
Misleading—due to being out of context	voting makes you lose your hunting license	AI might provide support tools
Innuendo and illogic	Anti-vax campaign	AI might provide some support but won’t solve

AI techniques are only useful as part of the toolkit. AI can support classifying messages by BEND objectives, or the perpetrators into types such as bots, trolls, and news-agents. The BEND framework and associated tools, some of which employ AI, can be used to assess how communications are spreading and measure the impact. For example, MemeHunter was used to identify an influence campaign from Russia engaged with a dismay objective that implied that compared to Russia, NATO was weak as the head of many countries defense were women, not a strong male military leader. This meme was spread by bots and humans alike.

## Research directions

Social cybersecurity is an exciting and emerging field. The BEND framework and so the associated theory and methods, BotHunter, MemeHunter and other such tools, are examples of the kind of work central to this area. However, much remains to be done. Indeed there are seven core research areas.

1. Social Cyber-Forensics: Social cyber-forensics is concerned with identifying who is conducting social cybersecurity attacks. Often the concern is with the type of actor rather than the specific actor. Further, this can involve cross platform assessment with the need to track down the source of information. New ways to track and build linkages at scale are needed.
2. Information Maneuvers: The key here is to understand the strategies used to conduct an attack and the intent of those strategies. Can we, for example, expand upon BEND to identify sets of maneuvers that are consistently used together or in a particular order to effect a particular impact? Improved abilities to detect maneuvers, and provide early warning that an attack is starting, are needed—particularly cross platform.
3. Motive Identification: The goal here is to understand what the perpetrators motive is. Why is the attack being conducted? Multiple motives have been seen. These include conducting influence campaigns: for fun, to create havoc, to polarize society, to alleviate boredom, for money, to polarize society, to market goods or services, to gain personal influence, and to generate community. There are likely to be other reasons as well. Being able to identify and track motive at scale and quickly is an important area for new research.
4. Diffusion: In this area the objective is to trace, and even predict, the spread of an influence campaign. A sub-aspect of this is to trace, and even predict, the movement of the components of a campaign such as people, ideas, beliefs, memes, videos, and images. Tracing the attackers and the impact of the attack across and through multiple social media is key. Live monitors that suggest when diffusion is about to explode, peak, and peter out. Improve theories of and methods for monitoring diffusion, particularly cross-platform are needed.
5. Effectiveness of Information Campaigns: The goal of this area is to quantify the effectiveness of the social cyber-security attack. This includes both the short term and the long term impact. It also involves creating improved measures of impact – such as polarization or mass-hysteria – rather than the traditional measures of reach such as number of followers, likes, and recommendation. While some measures cannot be done in real time, real time estimates of potential impact would be of value. New theories about impact and effect, as well as new techniques to measure effect are needed.
6. Mitigation: There are two related goals here. The first is to understand how a social cybersecurity attack be countered or mitigated. The second is to understand how communities can become more resilient to attacks. Many different avenues of research can be pursued here. Some examples are use of agent based models to assess the relevant impact of interventions, scalable techniques for teaching critical thinking for social media, and basic research on the characteristics of resilient communities. New empirical results, transdisciplinary theories, models and ways of measuring resilience and mitigation in this space are needed.
7. Governance: The objective here is to understand what policies and laws are needed so the people can continue to use the internet without fear of undue influence, so that an informed democracy can survive. This is a key area as it brings together issue of legality, rights, and education. This area needs to bring together all the diverse perspectives and diverse knowledge to develop actionable governance.

## Conclusion

**Fig. 3:**
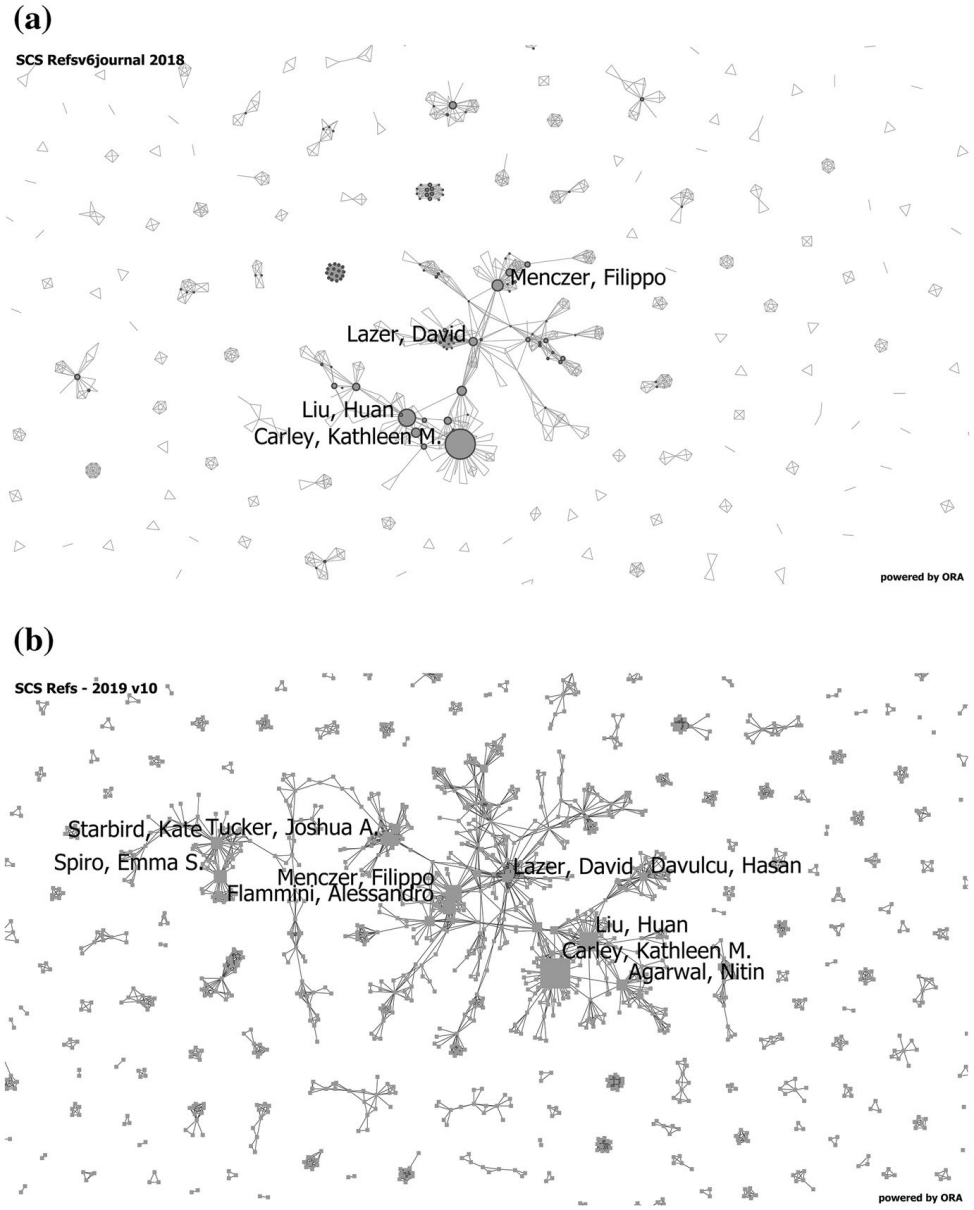
Evolving co-authorship network. The top image shows the central core in 2018 and the bottom image the central core in 2019. Each node is an author and the links are weighted by the number of papers those two authors co-authored

As noted, social cybersecurity is an emerging scientific and engineering discipline. While there are thousands working in this space, more research and more coordination of that research is needed. Work in this area began as interdisciplinary and is becoming transdisciplinary. Two words of caution. First, for individuals new to the area it is easy to come to the conclusion that little is known and that only a few individuals are working in this area. There are several reasons for this. First the research is spread across hundreds of venues with no one conference or journal being dominant. Second there is some research in most disciplines, but in each of the extant disciplines this is a fringe area. What we have found is that most researchers in this area do now know of others outside their own group. Often faculty in this area don’t even know of others in their own university. Greater outreach and ways of collaborating and coordinating across groups is needed. This is beginning to happen. In Fig. 3, the collaboration network based on who co-authors with whom, for 2018 and late 2019 is shown. As can be seen the central core has grown, and there are now links where none existed before. These growth is largely due to the Department of Defense Minerva program and the Knight foundation, both of which began to support research in social cybersecurity and to encourage collaboration.

Second, it is easy to think of this area as one where computer science and artificial intelligence will provide the solutions. Artificial intelligence solutions often aim at the easy things such as fact checking and currently require large levels of training data. But, this is a fast moving area where training sets are difficult to come by and are often out of data by the time they are created. It is easy to fall prey to stories that claim success because the mined extremely large data sets. But this fails to recognize that what is being discovered is the mean behavior and that most social change and social activity is on the fringe, and in the margins. What both approaches fail to recognize is that at its heart, social cybersecurity is about people as social beings, and it is people as social beings that are impacting and being impacted. To be sure artificial intelligence and data science are critical to this area; however, they should be in supporting positions not the drivers sear. What is needed is socially informed, social human being led computational social science.
